# Therapeutic genome editing by combined viral and non-viral delivery of CRISPR system components *in vivo*

**DOI:** 10.1038/nbt.3471

**Published:** 2016-02-01

**Authors:** Hao Yin, Chun-Qing Song, Joseph R Dorkin, Lihua J Zhu, Yingxiang Li, Qiongqiong Wu, Angela Park, Junghoon Yang, Sneha Suresh, Aizhan Bizhanova, Ankit Gupta, Mehmet F Bolukbasi, Stephen Walsh, Roman L Bogorad, Guangping Gao, Zhiping Weng, Yizhou Dong, Victor Koteliansky, Scot A Wolfe, Robert Langer, Wen Xue, Daniel G Anderson

**Affiliations:** 1 David H. Koch Institute for Integrative Cancer Research, Massachusetts Institute of Technology, Cambridge, Massachusetts, USA; 2 RNA Therapeutics Institute, University of Massachusetts Medical School, Worcester, Massachusetts, USA; 3 Program in Molecular Medicine, University of Massachusetts Medical School, Worcester, Massachusetts, USA; 4 Department of Biology, Massachusetts Institute of Technology, Cambridge, Massachusetts, USA; 5 Department of Molecular, Cell and Cancer Biology, University of Massachusetts Medical School, Worcester, Massachusetts, USA; 6 Program in Bioinformatics and Integrative Biology, University of Massachusetts Medical School, Worcester, Massachusetts, USA; 7 Department of Bioinformatics, School of Life Science and Technology, Tongji University, Shanghai, P.R. China; 8 Department of Biochemistry and Molecular Pharmacology, University of Massachusetts Medical School, Worcester, Massachusetts, USA; 9 Gene Therapy Center, University of Massachusetts Medical School, Worcester, Massachusetts, USA; 10 College of Pharmacy, the Ohio State University, Columbus, Ohio, USA; 11 Skolkovo Institute of Science and Technology, Skolkovo, Russia; 12 Department of Chemistry, M.V. Lomonosov Moscow State University, Leninskie Gory, Russia; 13 Department of Chemical Engineering, Massachusetts Institute of Technology, Cambridge, Massachusetts, USA; 14 Harvard-MIT Division of Health Sciences & Technology, Cambridge, Massachusetts, USA; 15 Institute of Medical Engineering and Science, Massachusetts Institute of Technology, Cambridge, Massachusetts, USA

## Abstract

The combination of Cas9, guide RNA and repair template DNA can induce precise gene editing and the correction of genetic diseases in adult mammals. However, clinical implementation of this technology requires safe and effective delivery of all of these components into the nuclei of the target tissue. Here, we combine lipid nanoparticle–mediated delivery of Cas9 mRNA with adeno-associated viruses encoding a sgRNA and a repair template to induce repair of a disease gene in adult animals. We applied our delivery strategy to a mouse model of human hereditary tyrosinemia and show that the treatment generated fumarylacetoacetate hydrolase (Fah)-positive hepatocytes by correcting the causative Fah-splicing mutation. Treatment rescued disease symptoms such as weight loss and liver damage. The efficiency of correction was >6% of hepatocytes after a single application, suggesting potential utility of Cas9-based therapeutic genome editing for a range of diseases.

The CRISPR (clustered, regularly interspaced, short palindromic repeats)/CRISPR-associated protein 9 (Cas9) system has emerged as a transforming genome editing tool^1–4^. Cas9:sgRNA recognizes the protospacer-adjacent motif (PAM) sequence and a complementary 20-nucleotide genomic sequence and induces double-strand DNA breaks, which are repaired by error-prone nonhomologous end-joining (NHEJ) or precise homology-directed repair (HDR)^2,5^. However, improvements to CRISPR delivery methods and HDR efficiency are required for therapeutic application of genome editing for disease gene correction.

The liver disease hereditary tyrosinemia type I (HTI) is a particularly suitable model for gene repair-based therapy because the repaired hepatocytes will expand and repopulate the liver^6,7^. In HTI patients, mutation of fumarylacetoacetate hydrolase (FAH), the last enzyme catalyzing the tyrosine catabolic pathway, leads to accumulation of toxic metabolites and severe liver damage^8^. The *Fah^mut/mut^* mouse model^6,8^ is caused by a G→A point mutation in the last nucleotide of exon 8. This causes skipping of exon 8 and truncated *Fah* mRNA. These mice can be treated with 2-(2-nitro-4-trifluoromethylbenzoyl)-1,3-cyclohexanedione (NTBC), an inhibitor of an enzyme upstream of Fah, to prevent toxin accumulation in hepatocytes^8^.

Several groups have also demonstrated *in vitro* correction of genetic disease genes including *CFTR* (cystic fibrosis)^9^, *Crygc* (cataracts)^10^ and *DMD* (Duchenne muscular dystrophy)^11^ in organoids or mouse zygotes. Adenovirus or adeno-associated virus (AAV)-mediated CRISPR-Cas9 delivery has been successfully applied to knockout genes in the mouse brain and liver^12,13^. Local delivery of Cas9:sgRNA complex in the mouse inner ear has also been reported^14^. However, all of these delivery studies have reported gene knockout rather than gene repair, a less efficient process requiring a DNA repair template^15^. Previously, our groups have shown that hydrodynamic injection of CRISPR-Cas9 DNA and a short single-stranded DNA HDR template can correct the *Fah* mutation in the mouse liver^16^. Nevertheless, hydrodynamic injection yielded a low correction rate of 0.4% of hepatocytes, and this method has been tested in only one human clinical trial^17^.

Adeno-associated viral particles have great promise as gene delivery agents^18^. However, AAV has a loading capacity size of approximately 4.7 kb, and the most commonly used form of Cas9 from *Streptococcus pyogenes* is difficult to fit in typical AAV constructs with efficient promoters^13^. Recently, a smaller form of Cas9 was developed, and shown capable of packing and delivery with sgRNA by AAV *in vivo*^12^. However, a repair template will require a second AAV vector. Moreover, concerns regarding potential for DNA damage remain if Cas9 is present for an extended period of time^14^. An alternative approach is the non-viral delivery of Cas9 mRNA, which would allow for short-term expression and, eventually, complete removal of the nuclease from the body. Lipid and lipid-like formulations have shown great promise as delivery agents for siRNA in a range of species, including humans^19^.

Here we report that systemic delivery of Cas9 mRNA by lipid nanoparticles and sgRNA/HDR template by AAV can efficiently cure *Fah^mut/mut^* mice. We observed an initial Fah correction in more than 6% of hepatocytes, suggesting that systemically delivered combinations of viral and nonviral CRISPR constructs may have utility for the treatment of a range of diseases.

To explore whether lipid nanoparticles can deliver Cas9 (*S. pyogenes* Cas9) mRNA, Cas9 mRNA was formulated with C12-200, a lipid-like material previously demonstrated to be capable of facilitating siRNA delivery in rodents and primates^20^, and associated helper lipids^21^ using controlled microfluidic mixing systems^22^. The Cas9 mRNA was chemically modified to reduce TLR (Toll-like receptor) responses^23^ (Supplementary Fig. 1a). These particles (termed nano.Cas9 hereafter) appear spherical in morphology with a textured interior under cryo-TEM (transmission electron microscopy) (Supplementary Fig. 1b). The mean particle diameter of nano.Cas9 is about 120 nm as determined by dynamic light scattering (Supplementary Fig. 1c). The particle size of nano.Cas9 was the same on day 0, 7, 11 and 18 (Supplementary Fig. 1d,e), indicating these particles are stable for at least 18 d in PBS. To test whether nano.Cas9 was functional, we used a 293T reporter cell line stably expressing a GFP reporter and a GFP-targeting sgRNA (sgGFP) (Fig. 1a and Supplementary Tables 1 and 2). Cas9-mediated frameshift NHEJ events will result in GFP-negative cells. 293T cells were incubated with 0.4 μg/ml nano.Cas9 and GFP signal was measured by FACS at 5 d. 77.1 ± 2.6% of cells (*n* = 3) became GFP-negative after nano.Cas9 treatment, suggesting that nanoparticle delivery of Cas9 mRNA can mediate genome editing in cells (Fig. 1b). To confirm that the GFP-negative cells were caused by Cas9 editing, we performed deep sequencing of the GFP provirus region from genomic DNA (*n* = 4). We observed insertional or deletional mutations (indels) surrounding the Cas9 cleavage site (Fig. 1c–e and Supplementary Table 3). Most indels are frameshift (e.g., 1 nt and 2 nt) mutations (Fig. 1e), which potentially cause loss of function of the GFP reporter. These data indicate that lipid nanoparticles can effectively deliver Cas9 mRNA into cultured cells. To compare the off-target effects of mRNA-mediated transient Cas9 expression with long-term viral Cas9 expression, we adapted a lentiviral Cas9 to mimic long-term Cas9 expression. Using a VEGFA sgRNA with well-characterized off-target sites^24^, we observed that transient Cas9 expression by mRNA delivery can substantially reduce off-target genome editing in cells (Fig. 1f–h).

Although lipid-nanoparticle delivery of siRNA to the liver has been reported, the systemic delivery of mRNA has only recently been developed^25^. To explore whether C12-200 lipid nanoparticles can systemically deliver Cas9 mRNA to adult animals, we first intravenously (i.v.) injected C12-200 lipid nanoparticles encapsulating β-galactosidase (β-gal) mRNA or Cas9 mRNA (Supplementary Fig. 2a). The size of β-gal mRNA is 3.3 kb whereas Cas9 mRNA is 4.5 kb, and the activity of β-gal protein can be detected by an enzymatic reaction. β-gal protein was detected in the mouse liver by immunoblot at 14 h after administration of a single dose (1 mg/kg or 2 mg/kg), and the amount of protein expressed correlated with the dose of mRNA (Supplementary Fig. 2b). To investigate whether β-gal is functional *in vivo*, we measured enzyme activity in the mouse liver^26^. The majority of the cells in liver sections stained positive in a β-gal activity assay (Supplementary Fig. 2c), indicating systemic delivery of mRNA to most of the cells in the mouse liver. To determine whether lipid nanoparticles can deliver Cas9 mRNA, nano.Cas9 (1 mg/kg or 2 mg/kg) was injected intravenously, and Cas9 protein in total liver lysates was detected by immunoblot (Supplementary Fig. 2d). To measure the half-life of Cas9 mRNA *in vivo*, total RNA of the liver was extracted and was measured by qPCR. The Cas9 mRNA was present in the liver at 4 h and 14 h but was significantly diminished at 24 h (*P* < 0.05), consistent with transient expression (Supplementary Fig. 2e). Nano.Cas9 (2 mg/kg) is well tolerated in animals, as indicated by intact liver histology, normal serum biochemistry and cytokine levels in plasma (Supplementary Fig. 3a–c).

To investigate whether nano.Cas9 can induce genome editing *in vivo,* we used the *Fah^mut/mut^* mouse model of HTI^6^. These mice possess the same G→A mutation in exon 8 as the common form of this human disease^8^. To enable repair of the *Fah* gene, we designed an AAV vector with a U6-sgRNA expression cassette and an HDR template (termed AAV-HDR hereafter), which contains a 1.7-kb sequence homologous to the *Fah* genomic region (Fig. 2a). We designed the HDR template to (i) correct the mutant “A” residue to wild-type “G” and (ii) introduce a “CC” motif in the PAM to prevent the recleavage of the repaired chromatid following HDR^27^ (Fig. 2a). Vectors were packaged using an AAV2/8 serotype, which can target the hepatocytes^8^. To optimize the delivery and assay regime, we identified the time course of sgRNA expression in the mouse liver. *Fah^mut/mut^* mice (*n* = 4 mice) were injected with 6 × 10^11^ genome copies of AAV-HDR, and sgRNA expression was examined at days 0, 3, 7 and 14 (Supplementary Fig. 4). We found sgRNA was already expressed at day 3, but its levels were more than tenfold higher at day 7 and day 14. Thus, to ensure maximal co-expression of all components, we injected nano.Cas9 7 d after AAV-HDR treatment. To explore whether the nano.Cas9 and AAV-HDR combination treatment can repair the Fah mutation *in vivo*, *Fah^mut/mut^* mice (*n* = 3 mice) were i.v. injected with AAV-HDR (6 × 10^11^ genome copies) at day −14, 2 mg/kg nano.Cas9 at day −7 and taken off NTBC water at day 0 (Fig. 2a,b). This AAV dose is comparable with a recently published study of AAV-mediated expression of human coagulation factor IX gene delivery^28^. Mice treated with PBS, AAV-HDR alone or nano.Cas9 alone served as controls. Nano.Cas9 + AAV-HDR treatment completely prevented body weight loss upon NTBC water withdrawal, whereas control mice rapidly lost 20% body weight and had to be euthanized (Fig. 2b). All the mice in the nano.Cas9 + AAV-HDR group survived 30 d after NTBC withdrawal. At 30 d after NTBC water withdrawal, serum biomarkers (AST, ALT and bilirubin) indicated that liver damage was substantially reduced in nano.Cas9 + AAV-HDR–treated mice compared to control mice (Fig. 2c). Immunohistochemistry staining detected widespread patches of Fah-positive hepatocytes (Fig. 2d).

To determine the initial *Fah* gene repair rate *in vivo*, we injected *Fah^mut/mut^* mice with nano.Cas9 and AAV-HDR and kept the mice on NTBC water for 7 d to prevent expansion of Fah-corrected cells (Fig. 3a). Up to 6.2% ± 1.0% hepatocytes stained positive for the Fah protein by immunohistochemistry in nano.Cas9 plus AAV-HDR–treated animals (Fig. 3b,c; *n* = 4 mice; see Supplementary Table 4 for percentage of Fah-positive cells, age and gender of each mouse). The number of Fah-positive hepatocytes correlated with the dose of nano.Cas9 and AAV-HDR (Fig. 3c). To investigate whether Fah splicing is restored in the liver, we performed qRT-PCR using primers spanning Fah exons 8 and 9 and observed that 9.5% FAH mRNA expression was restored without selection (Fig. 3d). Sanger sequencing of the RT-PCR bands in nano.Cas9 + AAV-HDR–treated mice confirmed that the corrected G nucleotide is present at the end of exon 8 (Fig. 3e).

To examine the efficiency of genome editing in the liver, we performed deep sequencing of the *Fah* locus in total liver genomic DNA. We observed an average of 24.1% indels at predicted sgRNA target region within nano.Cas9 (2 mg/kg) + AAV-HDR group (1.2 × 10^12^) (Fig. 3f and Supplementary Table 5) (*n* = 4 mice). The analysis of deep sequencing data confirmed a corrected “G-CC” pattern at the *Fah* locus in 0.81% ± 0.08% of the total liver DNA, in contrast to the AAV-HDR alone group, which contained no detectable “G-CC” sequences (Fig. 3g). We also designed a PCR approach to prove substitution of the transgene to complement the deep sequencing data (Supplementary Fig. 5a). We observed a clear band in livers of nano.Cas9 + AAV-HDR–treated animals, but not in control animals (Supplementary Fig. 5b). We sequenced the band and identified that the corrected sequence (“G-CC” pattern) is integrated (Supplementary Fig. 5c).

To explore AAV-mediated HDR at a second gene and compare the HDR and efficiency of nonviral and viral Cas9 delivery, we co-injected an adenovirus encoding Cas9 with AAV-HDR for Ctnnb1 (beta-Catenin)^29^. We observed ∼24% indels and ∼1.2% beta-Catenin–positive hepatocytes *in vivo* (Supplementary Fig. 6). Thus, at least for Ctnnb1, the all-viral Cas9 delivery did not significantly increase the HDR rate compared to mRNA delivery. CRISPR-Cas9 can cause indels at off-target genomic sites^24^. To determine potential off-target effects after Cas9 mRNA delivery *in vivo*, we performed deep sequencing at three of the top-ranking predicted off-target sites. Compared to indels at the on-target Fah site (Fig. 3f), <0.3% indels were detected at the assayed off-target sites in nano.Cas9 + AAV-HDR–treated mice, and these numbers were comparable with AAV-HDR–treated control mice (Supplementary Fig. 7 and Supplementary Table 6), indicating that Cas9 mRNA delivery has minimal off-target effects at assayed sites for *Fah* sgRNA.

To globally identify potential active off-target sites, we performed GUIDE-Seq^30^ in cultured mouse liver cells (Hepa1-6) by transfection of a pX330.sgFah plasmid. We observed a number of GUIDE-Seq oligonucleotide integration sites within the genome (Supplementary Table 7) but only the Fah target site and one other site (OT1, which is also the computationally predicted off-target site with the fewest mismatches in the genome; Supplementary Fig. 7) passed our stringent criteria for potential nuclease cleavage sites (Supplementary Table 8). These data suggest that there are likely not a large number of strong off-target sites for Cas9 programmed with the Fah sgRNA.

To more broadly investigate off-target editing, we performed targeted deep sequencing on nuclease-treated Hepa1-6 cells at the OT1 site as well as 11 additional genomic sites (GOT1-11) that displayed GUIDE-Seq oligonucleotide insertions (Supplementary Sequences). These additional sites did not meet our peak calling criteria, but we detected 2.4% and 1.8% indels at OT1 in two replicates (Supplementary Fig. 8a and Supplementary Table 9), consistent with OT1 being a Cas9:sgFah off-target site in transformed cells *in vitro*. To assess editing at these off-target sites *in vivo*, we performed targeted deep sequencing of the OT1 and GOT1-11 loci in treated livers. OT1 and several other assayed sites had modest indel rates, and none of these sites were substantially higher than the background indel rate in untreated livers (*P* > 0.05; Supplementary Fig. 8b and Supplementary Table 9). Our data, in particular the absence of significant lesions at the OT1 locus, indicate that the *in vivo* off-target lesion rate is low for sgFah in conjunction with mRNA delivery of Cas9.

Therapeutic editing has broad potential to treat a range of diseases through the permanent correction of genetic defects^31^. By combining viral and nonviral nucleic acid delivery, we report potentially therapeutically relevant formulations of CRISPR-Cas9 capable of inducing repair of a disease gene in adult animals. Systemic delivery of Cas9 mRNA by lipid nanoparticles, and sgRNA/HDR template by AAV, corrected a Fah mutation and restored Fah splicing in more than 6% of hepatocytes in the adult mouse liver, an order of magnitude improvement over that previously generated using high-pressure injection of DNA^16^. This treatment is well-tolerated in mice and fully rescued body weight loss and liver damage in tyrosinemia mice.

Our data suggest the efficiency of gene editing depends on the dose of Cas9 mRNA as well as sgRNA (Fig. 3f). To express sgRNA, we treated animals with up to 1.2 × 10^12^ AAV genome copies/mouse, a standard dose described in the literature^28^. It is possible to deliver sgRNA by non-viral delivery vectors to increase the level of sgRNA *in vivo*. A recent study demonstrated that the chemical modification of sgRNA improved gene editing *in vitro* when co-delivered with Cas9 mRNA^32^. Considering the higher barriers to successful *in vivo* delivery, chemical modification of sgRNA may prove to be a useful tool for systemic delivery.

The challenge of using non-viral vectors for CRISPR-mediated gene repair is to deliver Cas9, sgRNA and a repair template simultaneously *in vivo*. Especially the non-viral delivery of DNA into the nucleus *in vivo* with high efficiency and low toxicity remains difficult^19^. Thus, we combined non-viral delivery of Cas9 mRNA and an AAV vector with a sgRNA expression cassette and an HDR template. This allowed for short-term expression of the Cas9 nuclease, which provided efficient on-target genome editing, while potentially reducing off-target editing (Fig. 1h).

Previous work has reported on the use of zinc finger nucleases (ZFNs) for *in vivo* gene correction^31,33^. The CRISPR systems have a number of potential advantages over ZFNs, most importantly their flexibility and ease in adjusting target site^4^. A key feature of our system is the transient expression of the nuclease Cas9, through delivery of mRNA. This allowed for sufficient exposure to the nuclease for gene repair and reduced concerns associated with long-term nuclease exposure^34^.

A smaller form of Cas9 (*Staphylococcus aureus* Cas9), which fits into AAV vectors, has been recently used to knockout genes by NHEJ in the mouse liver. More than 40% indel formations in Pcsk9 locus was reported^12^. This number is higher than our report using mRNA delivery (∼24%). However, there are a number of differences between that study and the work reported here. First, Cas9 was delivered transiently using mRNA in our study, reducing the time window of exposure. Second, sgRNA was not co-delivered with the Cas9 enzyme, further narrowing the window of targeted cleavage. Third, template DNA was present in our work, providing for potential competition between NHEJ and HDR. Finally we expect that the efficiency of indel formations *in vivo* varies among loci. For example, less than 10% indels at the Apob locus in the mouse liver was reported in the same study^12^. Although NHEJ has been reported using AAV-mediated Cas9 delivery, HDR using viral delivery *in vivo* has not been reported. Here we showed more than 6% corrected hepatocytes, and HDR was confirmed by deep-sequencing analysis (Fig. 3). Our data suggest a model that only one corrected allele per polyploidy hepatocyte cell is sufficient to yield a Fah^+^ corrected cell, as suggested by the following calculation: 6% hepatocytes × 0.6 (fraction of hepatocytes in the liver)/4 (estimated average ploidy of hepatocytes) = 0.9% estimated DNA. This estimation is consistent with the 0.81% corrected “G-CC” sequence pattern observed by deep sequencing in treated liver samples (Fig. 3g). We consistently observed higher “CC” reads (about 2–4%) than “G” reads (about 1%) in treated samples. It is possible that our stringent “G-CC” pattern counting underestimated the HDR event. Nevertheless, considering the mixture of nonparenchymal cells (30–40% of total cells) and the polyploidy of hepatocytes in the mouse liver^35^, the deep sequencing data may be consistent with Fah^+^ hepatocytes. The ratio of positive stained hepatocytes seen by immunohistochemistry to HDR counted by deep sequencing is consistent with our previously published study^29^ where we used hydrodynamic injection of CRISPR-targeting β-catenin and a mutation template and observed ∼0.5% β-catenin-positive hepatocytes (0.5% hepatocytes × 0.6/4 = 0.075% estimated DNA) and ∼0.08% HDR by deep-sequencing reads^29^. An alternative explanation for this observation is partial gene conversion in spite of “G” and “CC” single-nucleotide polymorphisms equidistant to the Cas9 cutting site. It has been reported that partial HDR templates can recombine in the chromosome in classic DNA repair^36^, in ZFN-mediated gene correction^37^ and in Cas9-driven genome editing^38^.

Further improvement of HDR efficacy may be obtained by optimizing the HDR template design and inhibition of the NHEJ pathway^16^. *In vivo* delivery of Cas9 by mRNA may be suitable in both local and systemic situations for the treatment of disease. It is also possible to enhance Cas9 mRNA expression *in vivo* by optimizing its chemical modification. However, correction of 6% of hepatocytes following a single round of treatment without selection suggests that this approach may be suitable for the treatment of a range of metabolic liver diseases and hemophilia, where restoration of 3–7% normal functional protein can be therapeutic^39^.

## Methods

Methods and any associated references are available in the online version of the paper.

## Online Methods

### Animal experiments

All animal experiments were performed under the guideline of the MIT Animal Care and Use Committee. *Fah^mut/mut^* mice^8^ were kept on 10 mg/L NTBC water. Mice with more than 20% weight loss were humanely euthanized according to the MIT protocol. 1 or 2 mg/kg nano.Cas9 mRNA and 6 × 10^11^ or 1.2 × 10^12^ genome copy AAV8 were introduced in 8- to 10-week old *Fah^mut/mut^* mice (male and female) via tail vein injection. This AAV dose is ∼5- to 15-fold lower than the highest AAV dose approved for systemic gene therapy in human (3.3 × 10^14^/kg, RAC Protocol 1210-1189). To measure the initial repair rate, *Fah^mut/mut^* mice were kept on NTBC water.

### Cas9 mRNA nanoparticles formulation

Cas9 mRNA encodes the Cas9 protein with chemical modification of pseudouridine and 5-methylcytidine to decrease immune stimulation (Trilinkbiotech). nano.Cas9 was formulated with C12-200, cholesterol, C14PEG2000, DOPE(1,2-dioleoyl-sn-glycero-3-phosphoethanolamine), arachidonic acid, in a weight ratio of 50: 20:10:10:10 and Cas9 mRNA with a lipid:mRNA weight ratio of 20:1 using microfluidic method as previous described^22^.

### Construction of AAV vectors and virus production

AAV vector was constructed using Gibson assembly (Supplementary Sequences). AAV2/8 virus were prepared and purified by Boston Children's Hospital Viral Core.

### Liver histology, western blot, serum markers and cytokines

Mice were humanely sacrificed by CO_2_. Livers were freshly fixed with 4% PFA (para-formaldehyde) and embedded in paraffin. 4 μm sections were stained with hematoxylin and eosin (H&E) for pathology and with anti-Fah (ab81087, 1DB_ID: 1DB-001-0000324022 Abcam) antibody for immunohistochemistry^29^. The percentage of positive cells was measured at low magnification lens from >3 regions per liver in four mice per group. Western blot was performed as recently described^13^ using the following antibodies: Cas9 (7A9-3A3, Activemotif), beta-Gal (ab4761, Abcam)^40^. Blood was collected using retro-orbital puncture at the terminal time point. ALT, AST and bilirubin levels in serum were measured as described^16^. Cytokine levels in plasma were determined by Multi-Analyte ELISArray (Qiagen).

### Gene expression analysis and qRT-PCR

RNA was purified using Trizol (Invitrogen) and reverse-transcribed using a High-Capacity cDNA Reverse Transcription Kit (Applied Biosystems). Real-time PCR (qPCR) reactions were performed using gene specific primers (Roche 480). Data were normalized to actin.

### Cell culture, off-target analysis and Illumina sequencing

**Figure 1:**
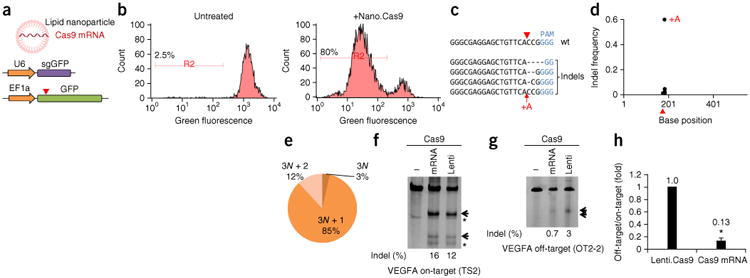
*In vivo* delivery of Cas9 mRNA mediates efficient genome editing in cells. (**a**) C12-200 lipid nanoparticle delivery of Cas9 mRNA into cells. 293T cells stably expressing both EF1a promoter-GFP and U6 promoter-GFP targeting sgRNA (sgGFP) were ncubated with Cas9 mRNA nanoparticles (nano.Cas9). Cas9-mediated frameshift NHEJ events will result in GFP-negative cells. Red arrowhead indicates the Cas9 cutting site. (**b**) FACS analysis shows that Cas9 mRNA generates GFP-negative cells. Gate R2 indicates 80% GFP-negative cells after nano.Cas9 treatment (*n* = 3). (**c**) GFP locus was deep sequenced in nano.Cas9 treated cells (*n* = 4). Shown are representative indels. (**d**) Distribution of indels. (**e**) Indel phase shows that most indels cause a frameshift. For example, 3*N* + 1 include 1-, 4- and 7-bp indels, 3*N* + 2 include 2-, 5- and 8-bp indels, and 3*N* include 3-, 6- and 9-bp indels. (**f**,**g**) Transient Cas9 expression by mRNA delivery can reduce off-target genome editing for a VEGFA sgRNA. 293T cells were co-transfected with Cas9 mRNA and pLKO. sgVEGFA (mRNA). 293T cells infected with lentiviral Cas9 were transfected with pLKO.sgVEGFA alone to represent long-term Cas9 expression (lenti). On-target (TS2) (**f**) and off-target (OT2-2) (**g**) indel rate was measured by surveyor assay at 2 d. Arrows denote indel bands. *, nonspecific bands. (**h**) Relative off-target/on-target ratio. The ratio in lenti.Cas9 was set as 1. *P < 0.01 (*n* = 3). Error bars, mean ± s.d.

293T (ATCC) cells were infected with lentivirus to stably express EF1a-GFP (Addgene 26777) and U6-sgGFP^41^. Cells were tested by MIT core facility for mycoplasma contamination by an ELISA-based assay, and confirmed negative. Cells were incubated with nano.Cas9 mRNA. GFP^+^ cells were counted by FACS. Off-target sites prediction was using http://crispr.mit.edu/^42^. For Figure 1f–h, 293T cells were co-transfected with 250 ng Cas9 mRNA and 300ng pLKO.sgVEGFA plasmid DNA using Lipofectamine 2000. 293T cells infected with lentiviral Cas9-Blast (Addgene 52962) were transfected with 300 ng pLKO.sgVEGFA alone to represent long-term Cas9 expression. Deep sequencing libraries were prepared from ∼1 ng purified PCR products using Nextera XT kits (Illumina). Libraries were sequenced on Illumina NextSeq500 (75bp, paired-end) and MiSeq (150 bp paired-end). Reads were mapped to reference sequences using bwa with custom scripts.

### GUIDE-Seq off-target analysis for SpCas9

We performed GUIDE-Seq using the corrected protocol^30^ with some modifications^43^ (described below) in the Hepa1-6 murine hepatocyte cell line (ATCC) to determine potential off-target sites in the Cas9-treated mouse liver. This cell line contains the wild-type splice acceptor site in *Fah*, such that there is a mismatch between sgRNA2 and the target sequence, which would should yield lower nuclease activity at the target site. 2.5 μg of pX330 (SpCas9 with sgRNA2) and 10 ng GFP were trans-fected into 3 × 10^5^ cells along with 40 pmol of GUIDE-seq oligos in a 6-well plate using Lipofectamine 3000 transfection reagent (Invitrogen) according to manufacturer's suggested protocol. 48 h post-transfection, genomic DNA was extracted via DNeasy Blood and Tissue kit (Qiagen) according to the manufacturer's suggested protocol. Library preparations are done with original adaptors according to protocols described by the Joung laboratory^30^, where each + and − strand library was separately barcoded for pooled sequencing. The barcoded, purified libraries were deep sequenced as a pool using two paired-end 150 bp MiSeq runs. The GUIDE-seq oligonucleotide sequences were removed from the Read1 and Read2 sequences and then mapped to the mouse genome using Bowtie. Reads containing the identical molecular index and identical genomic positions for the GUIDE-seq oligonucleotide insertion (based either on Read1 or Read2) were represented by one unique read. + or − strand peaks were separately identified from the mapped unique reads defined by the GUIDE-seq oligonucleotide insertion position using a custom pile-up program with a window size of 20 bp shifted in increments of 1 bp across the genome, followed by determination of the local maximum. The maximum peak value for every 20 bp region was defined for the + and − strand reads separately. Potential off-target sites were defined by loci with + and − strand peaks positions within 40 bp that have more than two unique reads on each strand. Neighboring + and − strand peaks that met this criteria were merged together using ChIPpeakAnno^44^. Regions spanning each peak start and end (± 50 bp) were searched for sequence elements that were complementary to the nuclease target site with NGG, NGA or NAG PAMs using CRISPRseek^45,46^. Only peaks that harbor a sequence with less than 7 mismatches to the target site were considered potential off-target sites. These regions are reported in Supplementary Tables 7 and 8 with the number of unique reads from the sense and the antisense libraries combined into the final read number.

### Statistics

*P* values were determined by Student's *t*-tests and one-way ANOVA with Tukey post-test using Prism 5 (GraphPad).

## Supplementary Material

ms #+ supp mats

## Figures and Tables

**Figure 2:**
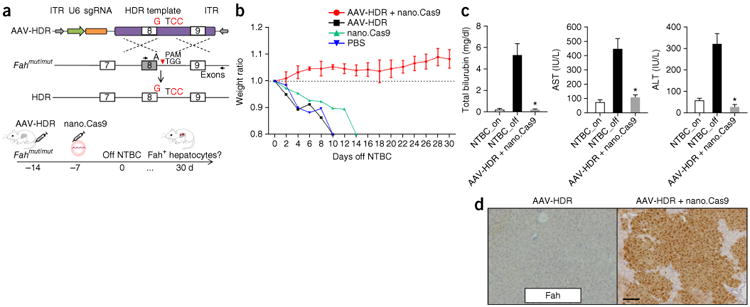
*In vivo* delivery of Cas9 mRNA and AAV-HDR template cures type I tyrosinemia mice. (**a**) Design of AAV-HDR template and experiments. G->A point mutation at the last nucleotide of exon 8 in *Fah^mut/mut^* homozygous mice leads to exon skipping of exon 8. A dual function AAV vector harbors U6-sgRNA and a HDR template (1.7 kb) with the “G” nucleotide to repair the “A” mutation. The “TGG” PAM was modified to “TCC” to prevent self-cleavage. Dashed lines denote homologous recombination. ITR stands for inverted terminal repeat. Black arrows indicate PCR primers for deep sequencing analysis. *Fah^mut/mut^* mice were injected with AAV-HDR and nano.Cas9 at indicated time points. Mice were kept off NTBC water at DO. Body weight normalized to pre-injection was monitored over time. (**b**) Delivery of AAV-HDR and nano.Cas9 fully rescues weight loss upon NTBC withdrawal (*n* = 3 mice). Error bars, mean ± s.e.m. (c) Liver damage markers (aspartate aminotransferase (AST), alanine aminotransferase (ALT), and bilirubin) were measured in serum *P < 0.01 (*n* = 3 mice) using one-way ANOVA. Error bars, mean ± s.e.m. (**d**) Fah^+^ cells after 30 d off NTBC. Scale bar, 100 μm.

**Figure 3:**
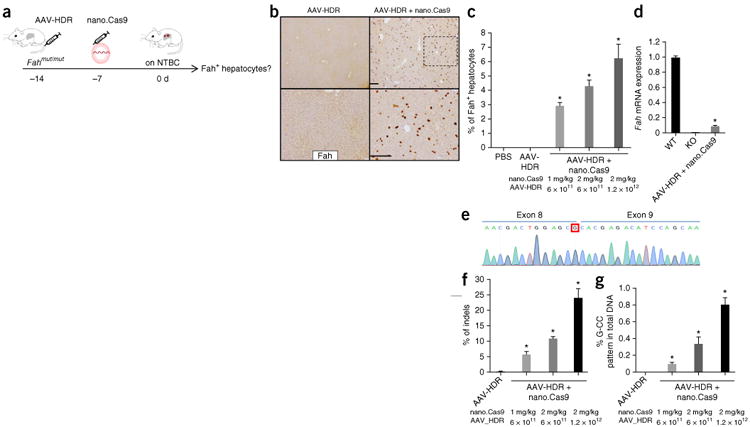
*In vivo* delivery of Cas9 mRNA and AAV corrects Fah mutation. (**a**) *Fah^mut/mut^* mice were kept on NTBC water and euthanized 7 d after nano.Cas9 treatment to estimate initial repair rate. (**b**) Fah immunohistochemistry (IHC). Scale bars are 200 μm for upper and lower panels, respectively. The lower panel of AAV-HDR + nano.Cas9 is a high-magnification view (box with black dashed line). (**c**) Fah^+^ positive cells were counted to determine the percentage. (**d**) Quantitative RT-PCR measurement of wild-type expression of Fah mRNA. (**e**) Sequence of repaired Fah mRNA in treated mice. QRT-PCR band of exon 8 to exon 9 spliced mRNA in (d) was sequenced. The corrected G nucleotide is circled. (**f**) Indels and (**g**) “G-CC” recombination pattern in total DNA from liver by Illumina sequencing. *P < 0.01 (*n* = 4 mice) using one-way ANOVA. Error bars, mean ± s.e.m.
