# Supplementary material for: Therapeutic genome editing by combined viral and non-viral delivery of CRISPR system components in vivo
Source: Nat Biotechnol. Author manuscript; Available in PMC 2017 May 9. (PMC5423356; doi:10.1038/nbt.3471)
Supplement: ms #+ supp mats [file NIHMS843247-supplement-Supplemental.pdf]

Fig. S1

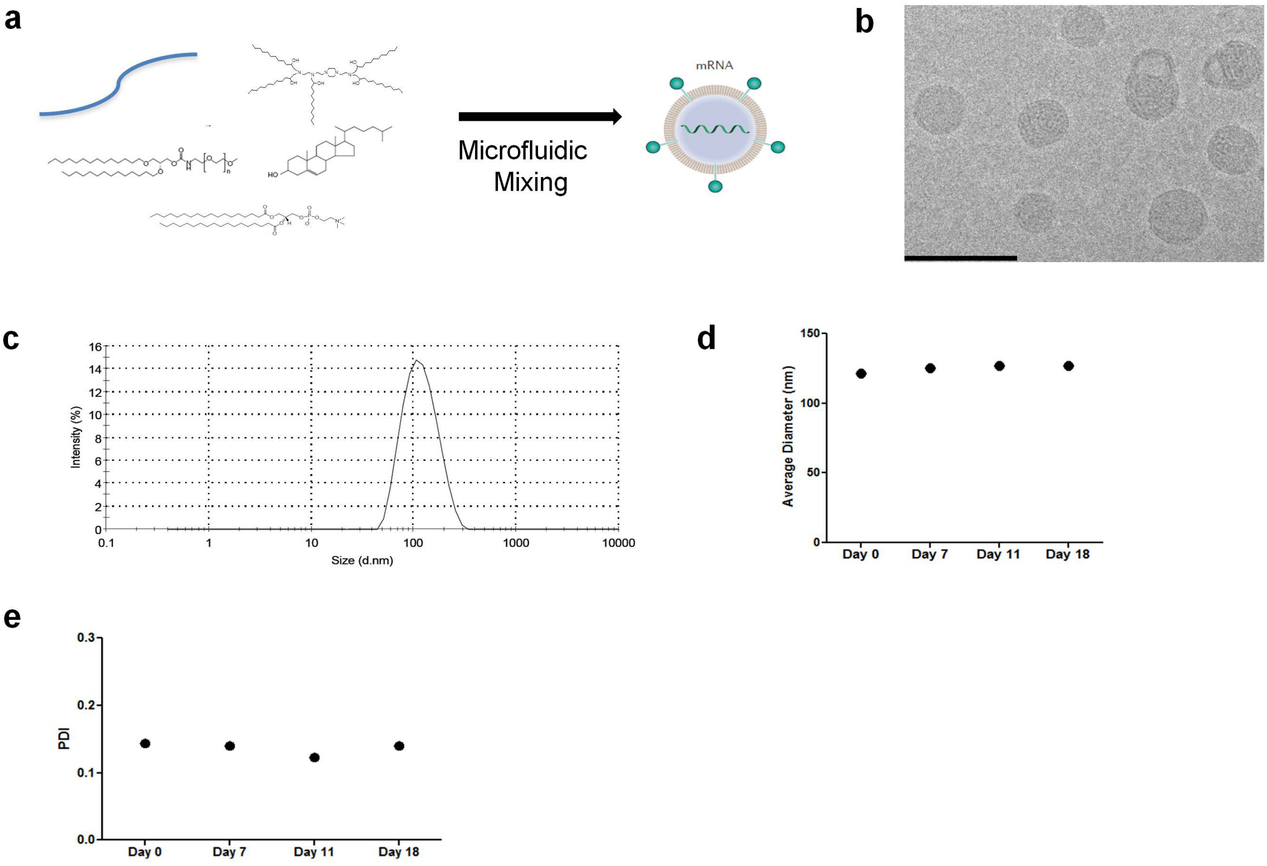

Supplementary Figure 1

Cas9 mRNA nanoparticles characterization.

(a) nano.Cas9 formulation scheme. Cas9 mRNA was mixed with C12-200, DOPE, Cholesterol, $C_{14}PEG_{2000}$ and arachidonic acid in a microfluidic chamber. (b) nano.Cas9 structure is characterized by cryo-TEM. Scale bar indicates 100nm. (c) Average diameter of nano.Cas9 was measured by dynamic light scattering. The size of nano.Cas9 (d) and the polydispersity index (PDI) (e) were measured 0, 7, 11 or 18 days after formulation and storage at 4˚C.

Nature Biotechnology: doi:10.1038/nbt.3471

Fig. S2

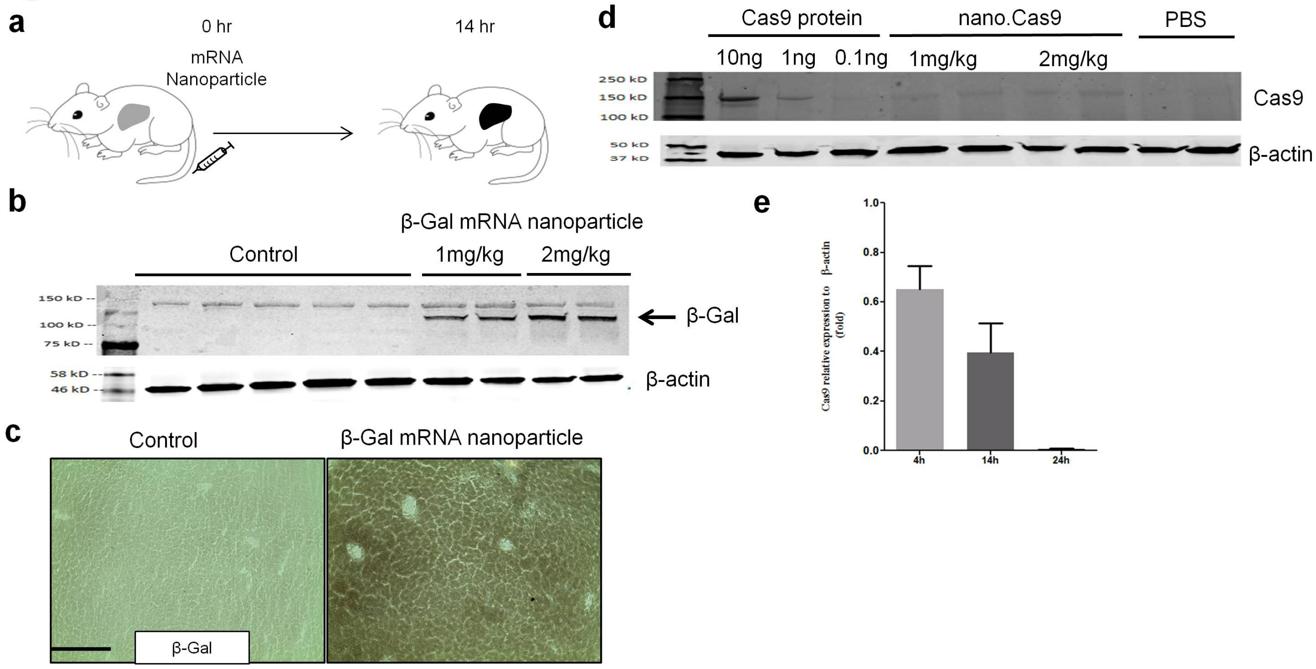

Supplementary Figure 2

The expression of proteins in mouse liver after mRNA nanoparticles treatment.

(a) C57bl/6 mice were i.v. injected with nanoparticles encapsulated with β-gal (b and c) or Cas9 mRNA (nano.Cas9, d and e), and livers taken. (b) The expression of β-gal protein was measured in liver lysate at 14 hours after injection. (c) The activity of β-gal in liver sections was determined by salmon-gal assay. Scale bar indicates 200 μm. (d) The expression of Cas9 protein was measured in liver lysate 14 hours after injection. 50μg negative control samples mixed with 10, 1 or 0.1ng Cas9 protein served as positive controls. β-actin served as a loading control in (b) and (d). (e) The Cas9 mRNA level in liver was determined by qRT-PCR at 4, 14, and 24 hours after injection (n=3 mice).

Nature Biotechnology: doi:10.1038/nbt.3471

Fig.S3

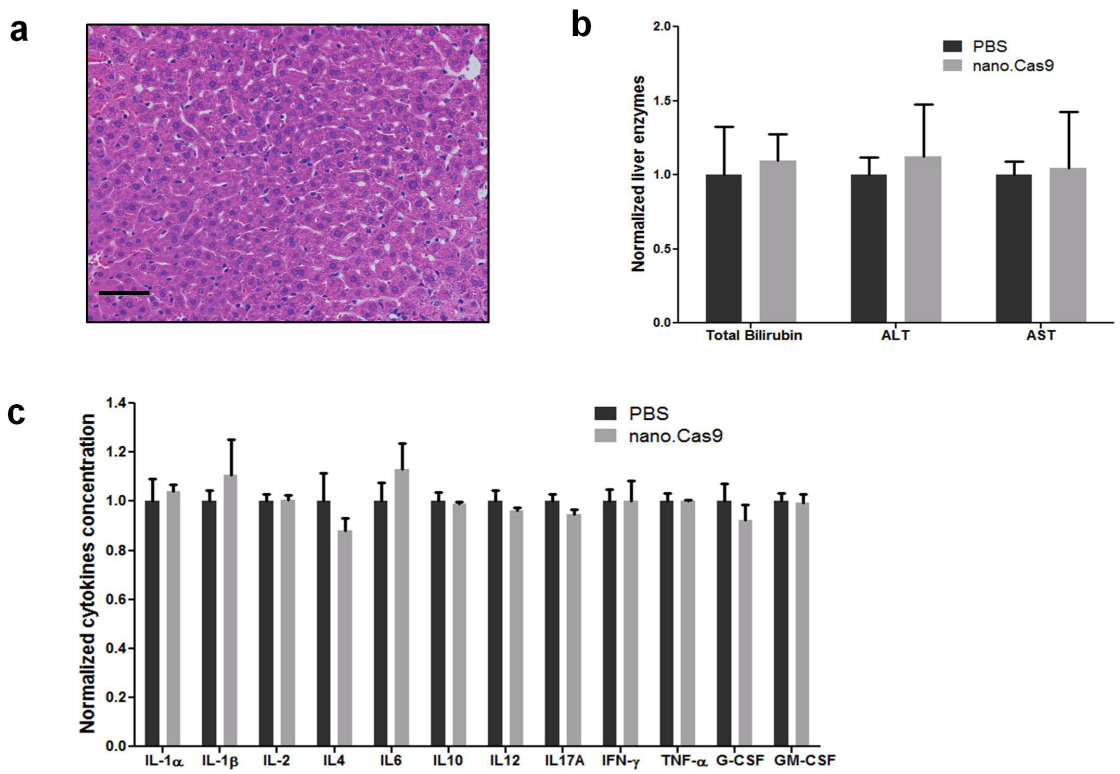

Supplementary Figure 3

Cas9 mRNA nanoparticles are well tolerated.

C57/Bl6 mice were treated with 2mg/kg nano.Cas9, and histology (a), the levels of liver damage markers (b) and plasma cytokines (c) were determined after 24 hours. Scale bar indicates 50μM. (n=4 mice).

Fig S4

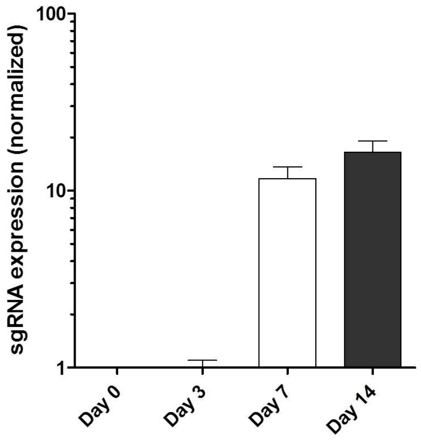

Supplementary Figure 4

The time course of sgRNA expression in mouse liver.

Mice were injected with AAV-HDR and livers taken at 0, 3, 7 and 14 days after injection. qRT-PCR was performed to determine sgRNA expression in liver. The expression levels were normalized to Day 3 (n = 4 mice).

Nature Biotechnology: doi:10.1038/nbt.3471

Fig S5

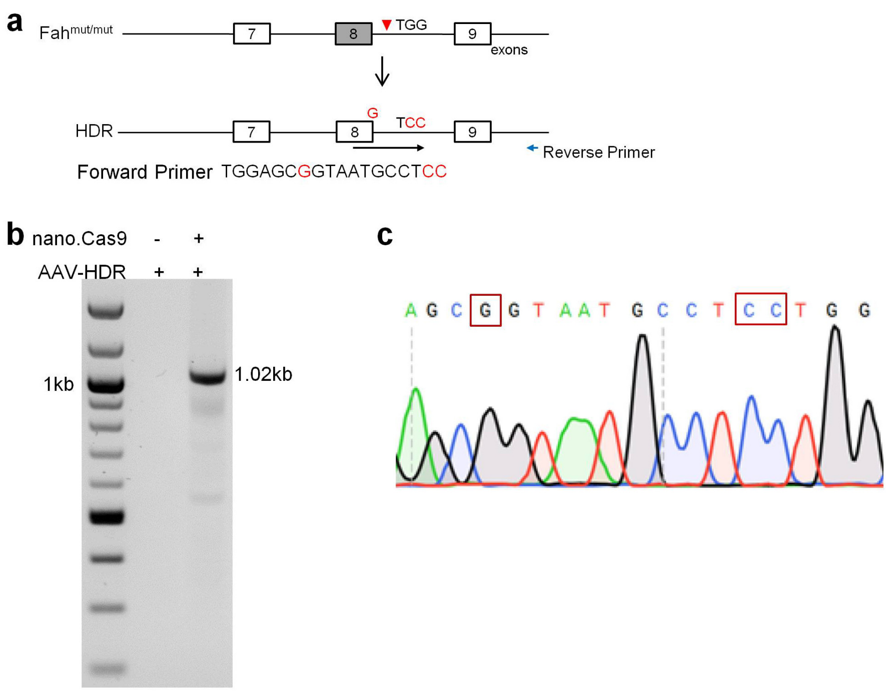

Supplementary Figure 5

A PCR approach proves substitution of the correct sequence.

(a) Design of the PCR primers. Blue arrow indicates the reverse PCR primer, which is outside the repair template. The sequence of the forward primer is presented, and “G” and “CC” in the corrected sequence are highlighted. (b) Genomic DNA of the liver tissue was extracted, and PCR was performed using the primers in (a). The predicted size of PCR product is 1.02kb. A representative sample from each group is shown (n = 3 mice). (c) The PCR product from (b) was cloned to a TA cloning vector and Sanger sequenced. The corrected “G” and “CC” are highlighted.

Fig S6

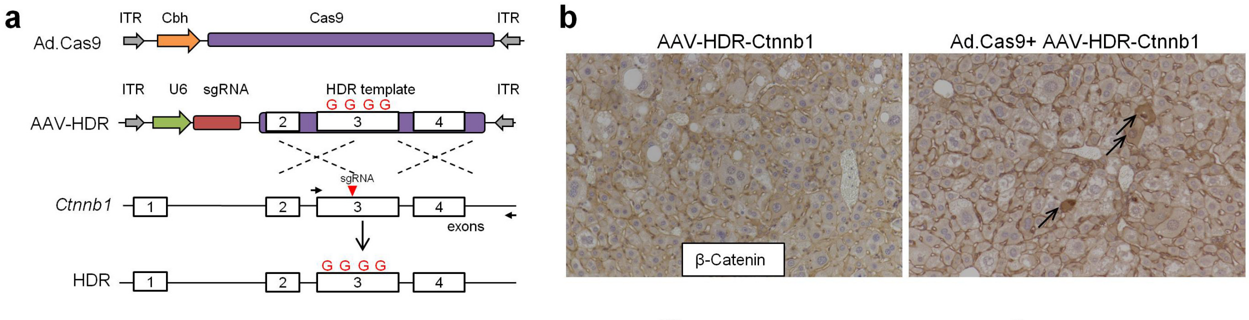

c

Mouse	Position	Ref	Indel	Ref	Indel	VarFreq
1	5	G	*/-A	1995	26	1.29%
	333	C	*/+T	5907	1650	21.58%
2	5	G	*/-A	1187	19	1.58%
	333	C	*/+T	5618	1898	24.88%

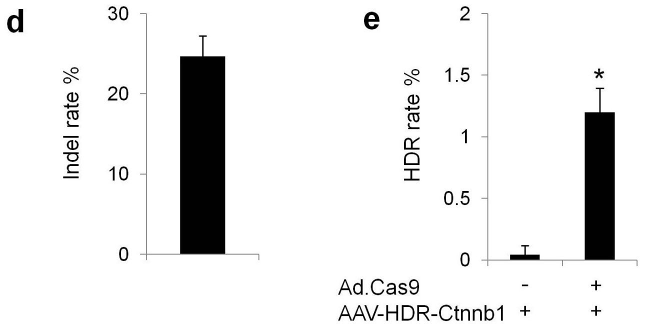

Supplementary Figure 6

Viral delivery of Cas9 does not increase HDR rate compared to mRNA delivery.

(a) Design of AAV-HDR template. Four "G" point mutations resulting in stabilization of β-Catenin are highlighted. Ad.Cas9 is an adenovirus expressing Cas9. (b) β-Catenin IHC. AAV-HDR-Ctnnb1 alone serves as a control. Arrows denote β-Catenin positive hepatocytes. (c-d) The Ctnnb1 locus in the liver total DNA of Ad.Cas9+ AAV-HDR-Ctnnb1 treated mice (n=2) were deep sequenced to measure indels. (e) β-Catenin positive hepatocytes were counted to determine the percentage of HDR. $P < 0.01$ (n = 3 mice). mRNA delivery of Cas9 yields higher rate of HDR for Fah (>6%).

Fig S7

a

```
                         PAM
ACGACTGGAGCAGTAATGCCTGG sgFah.2      Predicted off-target score
ACGACTGGAGCGGTAATGCCTGG wt Fah       49.2
ACAACTGGAGCAGAAATGCCAGG OT1          1.4
TCCATTGGGGCAGTAATGCCAGG OT3          0.8
ACGAATGTATTAGTAATGCCTGG OT4          0.7
```

b

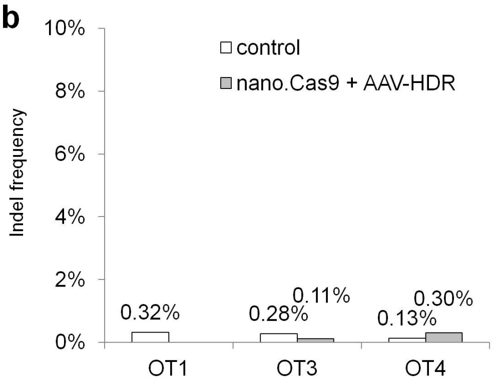

c

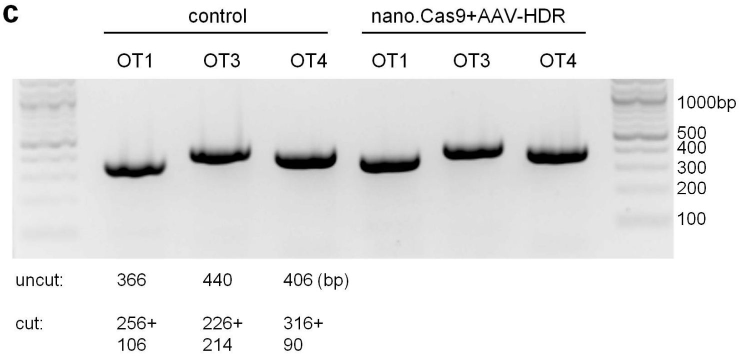

Supplementary Figure 7

Cas9 mRNA delivery has minimal off-target effects at assayed sites *in vivo*.

(a) Top ranking off-target sites (OT1, OT3 and OT4) for sgFah and the predicted score (Hsu et al, 2013). Mismatch bases are in red. Score for the wildtype sgFah.2 targets the mutant Fah which has one mismatch with wildtype Fah (wt Fah). (b) Indel frequency is low and is comparable between control mouse and nano-Cas9+ AAV-HDR mouse. OT1, OT3 and OT4 regions were PCR amplified from mouse liver genomic DNA and analyzed by deep sequencing. (c) Surveyor assay did not detect indels at OT1, OT3 and OT4. Predicted size of uncut and cut bands are indicated.

Fig S8

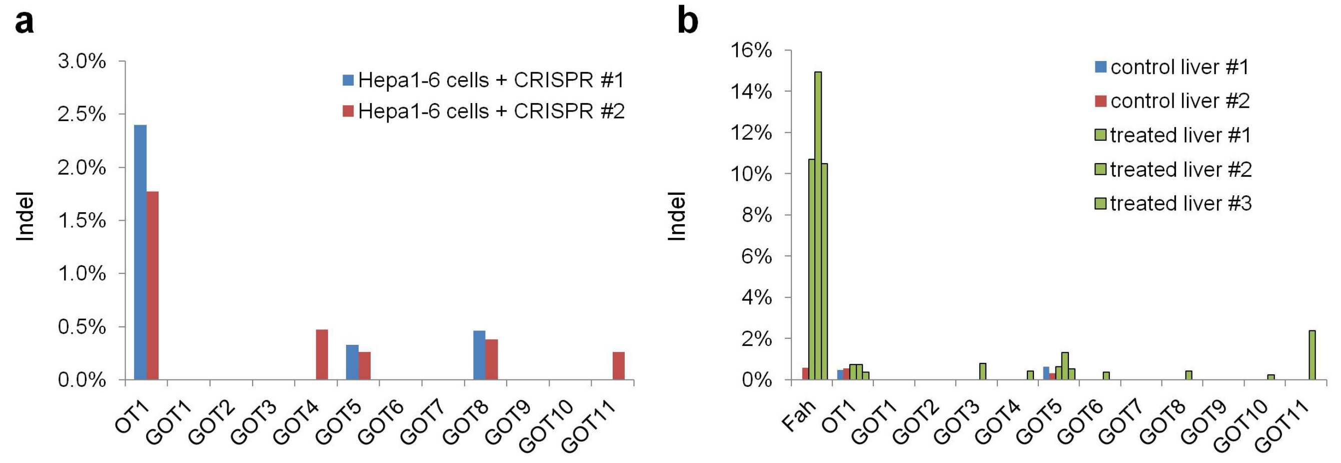

Supplementary Figure 8

Indel rate measured by deep sequencing for GUIDE-Seq off-target sites.

OT1 is the strongest off-target sites identified by GUIDE-Seq. GOT1-11 are additional genomic sites that displayed GUIDE-Seq oligonucleotide insertions. (a) Mouse Hepa1-6 liver cells transfected with pX330.sgFah.2. #1 and #2 are replicates. (b) Mouse livers treated with nano.Cas9 and AAV-HDR (treated) or control treated (control). Fah is the on-target site. See Table S9 for details.

Supplementary Materials

Supplementary Figure 1-8
Supplementary Table 1 sgRNA sequences.
Supplementary Table 2 Primer sequences.
Supplementary Table 3 Next-generation sequencing data for GFP reporter.
Supplementary Table 4 Percentage of Fah$^+$ positive hepatocytes following *in vivo* delivery of Cas9 mRNA and AAV.
Supplementary Table 5 Next-generation sequencing data for Fah indels.
Supplementary Table 6 Next-generation sequencing data for off-target analysis.
Supplementary Table 7 sgRNA2 GUIDE-seq +&- strand peaks (in a separated file).
Supplementary Table 8 sgRNA2 GUIDE-seq merged peaks (in a separated file).
Supplementary Table 9 Deep sequencing of off-target sites (in a separated file).

Supplementary sequences

Table S1. sgRNA sequences. An extra "G" is added for U6 transcription.

sgRNA ID	20nt sequence (5' to 3')	PAM	strand
Fah.2	Gacgactggagcagtaatgcc	TGG	+
GFP	gggcgaggagctgttcaccg	GGG	+
β-Catenin	Gctgtggtggtggcaccagaa	TGG	-

Table S2. Primer sequences.

ID	Sequence (5'->3')	Notes
Fah.F8	AGAGCCAATCCCCATTTCCA	QPCR primer with Fah.R9
Fah.R9	CGGGGAGATTGTGGTTCCAA	
Fah.R10	TGAAGGGGTGGGATATTGCA	Fah.FM+R10 for HR
FAH_PointM_F	CAGGGAAGTAATGCCAGGTC	Fah PCR primers
FAH_PointM_R	TGCATGGTATCACCCCTGTA	
FAH2-OT1F	ACTCACACTGTCATCCCTCG	FAH2 Off target site 1
FAH2-OT1R	GTTCCCACTACCACAATGCC	
FAH2-OT3F	TCTGGGGATTGGGTAGTGAC	FAH2 Off target site 3
FAH2-OT3R	GTCCTGGCCCGGATTATACA	
FAH2-OT4F	AAGGCATTGGAAGGGCTAAT	FAH2 Off target site 4
FAH2-OT4R	GCTAAACACTTGGGGCATGT	
EF1a-F	TCAAGCCTCAGACAGTGGTTC	PCR the GFP region for sequencing

GFP-R	TCCTTGAAGTCGATGCCCTT	
Forward P	TGGAGCGGTAATGCCTCC	Forward P+R10 to show substitution
QF	GCAGTAATGCCGTTTTAGAGC	qRT-PCR of sgRNA
QR	CGACTCGGTGCCACTTTT	

Table S3. Next-generation sequencing data of GFP reporter.

Replicate	Position	Ref	Indel	Reads Supporting Ref	Reads Supporting Indel	VarFreq
1	177	A	*/-GCTGTTCACCGGGGTGGT	6784	83	1.18%
	184	C	*/+A	2350	3649	59.77%
	185	A	*/-C	1846	105	4.51%
	185	A	*/-CCGGGGTGGTGCC	1846	26	1.12%
	185	A	*/-CCGGGGTGGTGCCC	1846	25	1.07%
	185	A	*/-CC	1846	43	1.85%
	185	A	*/-CCGG	1846	53	2.28%
	185	A	*/-CCGGG	1846	35	1.50%
	185	A	*/-CCGGGG	1846	33	1.42%
	185	A	*/-CCGGGGT	1846	26	1.12%
2	5	G	*/-C	3372	51	1.49%
	177	A	*/-GCTGTTCACCGGGGTGGT	6535	90	1.32%
	178	G	*/-CTGTT	6330	72	1.10%
	184	C	*/+A	2332	3512	59.05%
	185	A	*/-C	1772	135	5.90%
	185	A	*/-CCGGGGTGGT	1772	25	1.09%
	185	A	*/-CCGGGGTGGTGCC	1772	29	1.27%
	185	A	*/-CCGGGGTGGTGCCC	1772	25	1.09%
	185	A	*/-CC	1772	30	1.31%
	185	A	*/-CCG	1772	25	1.09%
	185	A	*/-CCGG	1772	52	2.27%
	185	A	*/-CCGGG	1772	35	1.53%

	185	A	*/-CCGGGGTG	1772	24	1.05%
	185	A	*/+T	1772	23	1%
3	183	T	*/-CA	5919	74	1.20%
	184	C	*/+A	2131	3713	62.41%
	185	A	*/-C	1593	100	4.84%
	185	A	*/-CC	1593	31	1.50%
	185	A	*/-CCG	1593	33	1.60%
	185	A	*/-CCGG	1593	44	2.13%
	185	A	*/-CCGGG	1593	40	1.94%
	185	A	*/-CCGGGG	1593	33	1.60%
	185	A	*/-CCGGGGT	1593	27	1.31%
	185	A	*/-CCGGGGTG	1593	31	1.50%
4	184	C	*/+A	2036	3684	63.18%
	185	A	*/-C	1554	109	5.36%
	185	A	*/-CCGGGGTGGTGCC	1554	24	1.18%
	185	A	*/-CC	1554	45	2.21%
	185	A	*/-CCG	1554	23	1.13%
	185	A	*/-CCGG	1554	42	2.07%
	185	A	*/-CCGGG	1554	29	1.43%
	185	A	*/-CCGGGG	1554	27	1.33%
	185	A	*/-CCGGGGT	1554	23	1.13%
	185	A	*/-CCGGGGTG	1554	21	1.03%
	185	A	*/+T	1554	24	1.18%

Table S4. Percentage of Fah^+ positive hepatocytes following *in vivo* delivery of Cas9 mRNA and AAV. Age and gender of individual mice are indicated as in Fig. 3c.

Cas9 dose	AAV-HDR dose	Age (weeks)	Gender	% of Fah^+ hepatocytes
1mg/kg	6e11	8.5	Female	3.50
	6e11	8.5	Female	2.69
	6e11	8.5	Male	3.18
	6e11	8.5	Male	2.24
2mg/kg	6e11	8.5	Female	4.89
	6e11	8.5	Female	4.18
	6e11	8.5	Male	4.90
	6e11	9	Male	3.19
2mg/kg	1.2e12	9	Female	6.21
	1.2e12	9	Female	6.53
	1.2e12	9	Male	8.43
	1.2e12	9	Male	3.78

Table S5. Next-generation sequencing data for Fah indels in the liver. Indels ≥ 1% are shown.

Name (mRNA dose)	Reference	Position	Ref	Indel	Reads Supporting Ref	Reads Supporting Indel	VarFreq
D15-193(1mg/kg, 6e11)	FahHR	239	A	*/+T	6378	249	3.76%
	FahHR	237	T	*/-A	7351	76	1.02%
D15-194(1mg/kg, 6e11)	FahHR	239	A	*/+T	6139	269	4.20%
	FahHR	237	T	*/-A	7246	118	1.60%
D15-196(1mg/kg, 6e11)	FahHR	239	A	*/+T	6441	207	3.11%
D15-197(1mg/kg, 6e11)	FahHR	239	A	*/+T	6077	342	5.33%
	FahHR	237	T	*/-A	7149	190	2.59%
D15-7095(2mg/kg, 6e11)	FahHR	239	A	*/+-T	6800	652	8.75%
	FahHR	237	T	*/-A	7403	188	2.48%
D15-7105(2mg/kg, 6e11)	FahHR	239	A	*/+T	6851	687	9.11%
	FahHR	237	T	*/-A	7463	140	1.84%
D15-7107(2mg/kg, 6e11)	FahHR	239	A	*/+-T	6828	588	7.93%
	FahHR	237	T	*/-A	7424	142	1.88%
D15-7109(2mg/kg, 6e11)	FahHR	239	A	*/+T	6921	596	7.93%
	FahHR	237	T	*/-A	7475	98	1.29%
D15-7088(2mg/kg, 1.2e12)	FahHR	239	A	*/+T	5523	1674	23.26%
	FahHR	237	T	*/-A	7255	255	3.40%
D15-7091(2mg/kg, 1.2e12)	FahHR	239	A	*/+-T	6544	921	12.34%
	FahHR	237	T	*/-A	7411	192	2.53%
D15-7096(2mg/kg, 1.2e12)	FahHR	239	A	*/+-T	5911	1464	19.85%

	FahHR	237	T	*/-A	7365	316	4.11%
D15-7097(2mg/kg, 1.2e12)	FahHR	239	A	*/+-T	5700	1572	21.62%
	FahHR	237	T	*/-A	7251	346	4.55%

Table S6. Next-generation sequencing data for off-target analysis of FAH2. OT1, 3, 4 indicate off-target sites 1, 3, 4. Shown are indels ≥ 0.1%.

Name	Reference	Position	Ref	Indel	Reads Supporting Ref	Reads Supporting Indel	VarFreq
control	OT1	144	T	*/-G	7938	11	0.14%
	OT1	179	A	*/+T	7970	14	0.18%
	OT3	188	G	*/-T	7853	12	0.15%
	OT3	233	G	*/-C	7880	10	0.13%
	OT4	194	A	*/+C	7957	10	0.13%
nano.Cas9 + AAV-HDR	OT3	188	G	*/-T	7856	9	0.11%
	OT4	194	A	*/+C	7949	15	0.19%
	OT4	350	A	*/+C	7958	9	0.11%

Nature Biotechnology: doi:10.1038/nbt.3471

Supplementary sequences

>Fah$^{mut/mut}$ reference sequence,FM+R10 PCR product, mutation and PAM are in red.
Exon is underlined.

CAGGGAAGTAATGCCAGGTCCTCAGGCAGCCCTAGTCCCTGGTTGAACTTTGAAAATATTTTCCCTTTGC
TCTGTAAGCCACAGTGACCCAGAGCATCGGGTCATCTAGATTCTTACCAACTTTCTCCATGGCAGGCTTT
CTTCGTAGGCCCTGGGAACAGATTCGGAGAGCCAATCCCCATTTCCAAAGCCCATGAACACATTTTCGGG
ATGGTCCTCATGAACGACTGGAGCAGTAATGCCTGGTGGCCCAGCTTCCTCTGATGTTCTGTTCTTAGGG
GCACACACAGGAGTTGGGTATGGGACAGGAGGCCTAAGTACTACAGGGGTGATACCATGCAGACTTCTGA
CTCTGTGGGTGTGGGGCAGTCACAGCTTCCCTGAGTAGCTTTCTCATAAGTGGAAGGATGGAGCTGACAG
AACCTAAAGCTTTATCAAGCCCTACACACTCCACTCACTGTTGCCAGCACCATCAGCTGCTGATGAAGAG
CTGTACATGGAGGGATTTTAGTCAGTGGCTGAATTGAGCCTTACTATTCATCAGCCCAGCATCTGAGTCC
CAGCTCTACACCCTGATACCATGTGTAAGAGGAAGTGGGTCCAGAAATCTCAGGAGAGGGCCTTCCCCCA
TTCAGGCCCTTGGTAGGGACTGTCCTGTCATCCTTCATTGTTCCCTAGCACAGCACCTCCAAGGACCTCT
GTCTTCATACTGAATACCTTTCTGTTTGTTCCCTGGGAGATCTGGTTGGCCAGAGCCATGTAGAGAGGTC
CATGTAGGTCAAGGACAGGAAGCAGCTTGGAGTGAGTCTGTAAGCCACGGTGAGACCTAAGCTTGCTGTC
TTTTCATAGCACGAGACATCCAGCAATGGGAGTACGTCCCACTTGGGCCATTCCTGGGGAAAAGCTTTGG
AACCACAATCTCCCCGTGGGTGGTGCCTATGGATGCCCTCATGCCCTTTGTGGTGCCAAACCCAAAGCAG
GTAAGCACCCTTCTGCTGAAAACTCTGCTCTAAGTTCCGGCTTCCGCCCTTTCCTGTTGCCCTCTGATAC
TGAGACATGGTGATGCTTGGTGGATCCTGCATTTTGTTTTGTGCAATGGCTCCCTCTAGAGTCTCATTTG
CAGAATGCATTTTGGAATAGAGGCTATAAGGGAAAAGGTGTGTGTGTGTGTGTGTGTGTGTGTGTGTGTG
TGTGTGTGTGTGTGTGTCCAACAGGGTTCCCAGTGCAATATCCCACCCCT

>Fah HDR reference sequence sgFah.2

CAGGGAAGTAATGCCAGGTCCTCAGGCAGCCCTAGTCCCTGGTTGAACTTTGAAAATATTTTCCCTTTGC
TCTGTAAGCCACAGTGACCCAGAGCATCGGGTCATCTAGATTCTTACCAACTTTCTCCATGGCAGGCTTT
CTTCGTAGGCCCTGGGAACAGATTCGGAGAGCCAATCCCCATTTCCAAAGCCCATGAACACATTTTCGGG
ATGGTCCTCATGAACGACTGGAGCGGTAATGCCTCCTGGCCCAGCTTCCTCTGATGTTCTGTTCTTAGGG
GCACACACAGGAGTTGGGTATGGGACAGGAGGCCTAAGTACTACAGGGGTGATACCATGCAGACTTCTGA
CTCTGTGGGTGTGGGGCAGTCACAGCTTCCCTGAGTAGCTTTCTCATAAGTGGAAGGATGGAGCTGACAG
AACCTAAAGCTTTATCAAGCCCTACACACTCCACTCACTGTTGCCAGCACCATCAGCTGCTGATGAAGAG
CTGTACATGGAGGGATTTTAGTCAGTGGCTGAATTGAGCCTTACTATTCATCAGCCCAGCATCTGAGTCC
CAGCTCTACACCCTGATACCATGTGTAAGAGGAAGTGGGTCCAGAAATCTCAGGAGAGGGCCTTCCCCCA
TTCAGGCCCTTGGTAGGGACTGTCCTGTCATCCTTCATTGTTCCCTAGCACAGCACCTCCAAGGACCTCT
GTCTTCATACTGAATACCTTTCTGTTTGTTCCCTGGGAGATCTGGTTGGCCAGAGCCATGTAGAGAGGTC
CATGTAGGTCAAGGACAGGAAGCAGCTTGGAGTGAGTCTGTAAGCCACGGTGAGACCTAAGCTTGCTGTC
TTTTCATAGCACGAGACATCCAGCAATGGGAGTACGTCCCACTTGGGCCATTCCTGGGGAAAAGCTTTGG
AACCACAATCTCCCCGTGGGTGGTGCCTATGGATGCCCTCATGCCCTTTGTGGTGCCAAACCCAAAGCAG
GTAAGCACCCTTCTGCTGAAAACTCTGCTCTAAGTTCCGGCTTCCGCCCTTTCCTGTTGCCCTCTGATAC
TGAGACATGGTGATGCTTGGTGGATCCTGCATTTTGTTTTGTGCAATGGCTCCCTCTAGAGTCTCATTTG
CAGAATGCATTTTGGAATAGAGGCTATAAGGGAAAAGGTGTGTGTGTGTGTGTGTGTGTGTGTGTGTGTG
TGTGTGTGTGTGTGTGTCCAACAGGGTTCCCAGTGCAATATCCCACCCCT

>GFP reference sequence. PAM is in blue.

tcaagcctcagacagtggttcaaagtttttttcttccatttcaggtgtcgtgaggaattagcttggtact
aatacgactcactatagggagacccaagctggctagttaagcttgatatcgaattcctgcagcccggggg
atccaccggtcgccaccatggtgagcaagggcgaggagctgttcaccgggggtggtgcccatcctggtcga

gctggacggcgacgtaaacggccacaagttcagcgtgtccggcgagggcgagggcgatgccacctacggc
aagctgaccctgaagttcatctgcaccaccggcaagctgcccgtgccctggcccaccctcgtgaccaccc
tgacctacggcgtgcagtgcttcagccgctaccccgaccacatgaagcagcacgacttcttcaagtccgc
catgcccgaaggctacgtccaggagcgcaccatcttcttcaaggacgacggcaactacaagacccgcgcc
gaggtgaagttcgagggcgacaccctggtgaaccgcatcgagctgaagggcatcgacttcaagga

>OT1 PAMs is in blue.
actcacactgtcatccctcgcttttgtgtggattctgggatccaaactcagtcttcttgcttttatggta
gttgccttgccgagtgagtgcctctcagttcctggcatttctgctccagttgtacaatccttcctgctgt
ttctgatctggaggcgttagagctgcctcccctgctttatttgtcctgcccgggctagtttggtgtttcc
tgactgttcttaattggctttcaagtcatgtgacctagactcctgatagaaataacccaaggaaggagcg
tttatttggactgacagtttggagggggtttggtccatagtcacttggttccatgctgttgggcagggca
ttgtggtagtgggaac

>OT3
tctggggattgggtagtgacttgcccagcagaactaaaagtattttaaccattccttatttcttcttaa
aacatttctataaaaaagaaatggcatatgccttattattccctttcacgaggaacatattagaaatcag
ctcctttggctttggtgttttcaagatagccatccgtgagcttggctgttttcccagttctgagtgatgg
cagtcctagacctggcattactgccccaatggatcagtacttgatgagtcgccctagaggtaaagttggt
tctgggtgtgagcgctccccgtgtatcttaactgtcacaattcactgttttaagctggttgagtgttgag
ctttttctgaaccctctcaagagctagatctgggtagacacaggagtgattcagacccaccgtgcacact
tgtataatccgggccaggac

>OT4
tgcacaagaggggtacaattagggggaatcaatataaccatccttttacactcttccttgccactttcca
tttacgaatgtattagtaatgcctggcagaatggctttttaactaaaggaagattagtggtaagagcca
aagtgttccctaaaacacacatctctgggcaagtgcactgtgccaccagacagaagcactgataaggtta
agacaacctaaaccccaggaaatctcagaattgagaatggtagttatgaggacagctctctgccaaacta
tctgatctggaagaagtaaccatgacacagtactgagaggaccatgacaattagtcacatagtataaaaa
cctggagttttctatgataatgaggtatctagacatgccccaagtgtttagccaat

>GOT1: Primers and GUIDE-Seq peaks are underlined.
GAGGACCTGGTTCCTTTCTCAGTCTTCACTGCTGGGAGCTGCAGGGTGTCTGCTCCGTTTGGGCAGGCC
AGAGTCAGGAGCAGTGGGCACCCGTGTCTGCTATAAGCCTGTGCAGGACCTAGGCCGGGAGGAGTGG
AGCCCCAGCCTATAGCGGTGTGGCTCCTGCACGAGAGAGCCTGGTTAGTCTCCTGTTCACACCAAAGAT
GGTGTGGGTAGAGGCACCGTTTATCAGAATCAGCTCAGCCTCAGAAAGGGTGGATCTAGGTCATGAGG
GACTTCCCAGCTATCTGAATTATGATGCCTTGCCAGAGGAGGTAGATGAAAAGCCTCCTCCACACGGAC
AGTGCAAGGGAGACGTTATTTGAGGTTGACTAAAGGCAGTCATTTCTTTTGGTACATGAGAATAGACAT
TGGGGTACATTTCAAGGTCTGGGTACTAGCTCCATGGTAGAGAGCTTGCCTGGCTTACCGAGGACCTGG
TTCCTTTCTCAGTCTTCAC
>GOT2:
CTTAAACTCATTGGCACAGGGGGGAAATTTATATCTCACAAATGGGTAAAATTGTTGATTACTTTTCTACT
CTCTGTGCATAGAGCTTTCCAGCACTAGGAAAGTTAGCCAGTGATGATAAAGCCAGGTCAGTCTCAGCT
TGATTTCTCTATGTGCCATGATTGAAGGATGTGGTGTCTTTGGCAATAGGGTATCACCATTAAGTTCTGA
AGGGTTACCTAGAGATATGGTAATATTTTGTAATGTTTTTGGATTCCATTGACCAACAACCAGAAAAGAC

Nature Biotechnology: doi:10.1038/nbt.3471

ATACATATACCTGGCACTGTATATTTGGTGGTGTATAGTTTCTGGGAGACATATTGTCTTCCATTATGGG
GTAAGTGGATCAAGCACCTCAACATAAAACCACATACATTGAATCTAATAGAAGAGAAAGTGAGAAAG
AGCCTTAAACTCATTGGCACAGGGGGGAAATT

>GOT3:

ACTAGAGACTCATCACAGGCAATTCCAAGAATGTTGTTTATCCAAGCCCTACAGGACATTTGGATAAACC
CCTCAAGGCTCAGGGGACATTTCAGAAGGGGTTGGGATGAATATAGAAAGTCAGAAGACAGGAAGAA
GGGCTGCAGAACCCTAACTCCTAGACAGGACACAGACAGCATGGTAATCATGAACTCACAGTAGCTACA
CCTACTGGCACTGAGCCCTGCATACCAGGGCTCTCTTACAGTCATCTAAGGATAGGGAACAGGCTCAGA
AGGTCCTATCATTTACTGCTGAACTACTGGCAACTAAAGGAGGAAGTGTAAACACCAGCTGTCAGCCAC
TGGTGAGCCCACTAGGTGCTAATGTATAG

>GOT4:

TGAAGGCAGTTACTGAGCCAAATAGTGGAGGGAATACGGGCAGAGAGTCTGACCTGGATGGATAGTTA
GCCATTATCCACCTGGCTGCATACAAGGCCTCAGCACTATTATTTTTCCTATTTAAAAAAAAAAAAAAAG
AAACGTAATTCGTACATACACAAAAATGGACCCTTTTGCACATAGCTGAGGTATTTTTTTAAGCCACATA
AAATTATATAATCGCAGCCAGGCTTAGTAATACACACCTGTATTCCTGTAATCCTGGCACTTGAGAGGCA
GGGGCAGGAGGATCAAGAGTTCAAGGTCATCTTCAGTTCTGTGGCAAGACTAAGGCCAGAGCAAGTTT
CCTGAGACTTGCCTCAAAACCATCAGCAAAAGATAAAGAGCCATTGTGATGGGGAATTCTCAA

>GOT5:

ACATTCACCATGAAAACCAGATCCAATACATCAGATATTGGGGACATCCATACATTGGGTGACTGGGGA
CATTTCCCACACTAGATATTCAGTCTGAGGCTTGGCCAGTGGAAGGAGCCCTATTGATATTAATGCCAGA
GTGCTTCATGGAGAGACTGTCTCAACAGAGGAGCAAGCCTGGGTAGGTAGACCAATAATAGCCAGTAT
GGTTATGATATGCCTATGAGTCCTCATTGCTTAAATCTCCTGCTGTAAAAGCAACAGATAACCAGGATCT
CACTTGGCATCCCTTTTGGACCTACTACCATGTCCATGCTTAATCCATGGCTCTAATAGACTACTCCTAGC
CTATCTTCATGTGCGCAATGAGGGTA

>GOT6:

CCAGGAAAAACTCTTGGGGACATGTAAGTCACAGAACCGATTTCCTAAACAGATATTCCAGACTGGCCT
GAAACTCCATGGAAAGCTGGGACAGCCTGTAACCTTTGACCCCAAGTGCTGGCATCACCAGCGTGGCTG
GCTGGCTTATGTGGTTCTGCGCACTGAACACAACACTTCCTGGATGCAAGGAGAGCAGCTGTGAACTAA
TCTCCATCCCCACAGTGGTCCGTTTGCTGTTTTTTGACTTCTGCTTATTTGGCGGTAAGAAGGGAAAACAA
AGCCTTATCCGTGTAAAGCACTTAGTCCTGACGTAATGTCATTTCGAACAGTAAAGACTAGGCAGGAAA
CATACAACGAACACAGGCTTAACCTCACACAAGAAACAGTGTGCCATTCGCTGTTCTATTGGCAGATG

>GOT7:

CAAAAGAGAACCAGAGGGTAGCATGTTCATCCAGGAAGGAAGTACTGACGAGGCCTGTACCTGTCACC
TGCCCTGAGGCCCTTCCCAAGTGGCCTTCAATTCAAGGTGGCTCCAGTTTTGTCCAGTTCCTATGTCCTTC
CTAGGAGCTGTTGTTAACCGTCCCATGTGGTTGTGGGGTGGGGTGGAGCCCTTTGGGTTATGAAAAGG
CAGAAGAGCAGGCCTGGGTTAGGATCATTGCAGAGGAGTGTTGCCCTCATTGGGCCTGTCCCAGGTGA
TACGCATCTTCCCAGGCCTGGCAGTGAAGGATCACCTGCAGATCCCACACACAACACAGGGATGTAGGG
AAATCAAACAGTACGGAAGATGTGGTAGCACTGTGCAGAGCCCGCACTACCCAGGGAAAGAGG

>GOT8:

GTTAAATGTTGTCCTGAGATATGGGGGAAGAAACCTAATAAAACCTGATTTAAAATAGGAGCTAGGGAA
AGGTCTTCCCACTTTAAGTACAAACAAAAGTCAGCTTGTGTGGTAGGGGCCTAGAGTCCAGGGTAAGCA
GCAGCTTGTGTGAAGACCCTGAGCAGAAGCAAGCATGGCTTTTCCTGGACCACTTAGTGGCCTACAGCT
CAGTAAGTTCACTTGTCATTGCTCTGACACAGTGCCTGAGGAAGTGAACTTACAAAGGGGAAAGCTTTC
CTTTTGCTTACATTTTCAAAGTTTTCTGAGTATGGTTACTCGGCCCCGTGGCAGCAAAGCACATTAGGATA

AGAACCCAAGGCAAAGGAGACCCGCTCACCTCGTGGTGCTCAGGATGCAAGGAGGAAAGAGACAGGA
AGAGATCAGGATCCCAATATCCCCTTCGAGGGCTCTTTCCCAAGGACCTAATTTCCTTC
>GOT9:
ATAACCATCACTCTAATCCCAGGACATTGTCAGTCTCTGAGGGAACCCTGTATTTCACCCTTCCCCCAACC
TTGGCAGCCACTAGTCAGTTTTATATCTCTGGATTTATCTGTCCTGGGTATTCATATAAAGAGAACCATGA
AGTACCTGGGCCTTCATCTCCAGCATCTTTCACTTAGTATTATGTCTTCAAGGTTCAGCCATATTGTGGAA
TGCTGCAGTGCTCTATCTTTATTATTTAATCTTTATTTCTTGGATGCCTGTCTTATAGTCTGTTGCTCAGTTC
ATCAATTGTTGTATATATAAGTTGATTCTGTTCCTTATTGTAAGCAGTGCTATTTTGAATATTCAGGGTAG
ATTTTTGTGAACATGTGAGCTTATACTTAGGTTCTGATTACTTTAAAGAGGATTAGAGGTAGTGGATTAT
TGCTATTGCATGTACA
>GOT10:
AATGCTCAGGACAGGTCACACAATGTCAAGTGAAACCGAGACGTTCGATTTTCAGGATGTTCTGTCATT
GACCCTCTCTGGCATCCTGGTGAACTCAAGTGCTTTCTGGAGTTTTTTCAGCCCCAGAAATCCATGGGGT
GGCCACACTGTGTACATATGCACACGGTGACCCTATCATTTGCAGTAAAAGGATAATGGCCTTATACTCT
TGACCCAAGATGACCTGCCCAAGTGTCCTGTGGGGAAAGAGAGGGGTCATGAGAGGTCAGCAGAAAG
TCACCTCCATGGTTATGTCCCATGACTCCTTAGGCATCTGGAGAACAGGGGCCAAGCCCCCTCCTCATAG
GGCAGTTGGGGGATTCAGATAAATACCTACCCCAGGAACACTTCTCTGTGACCGGAGGCCCACCTGCAC
ATCAGTTATGATTCAGCACCAGAAAAGGGGCAGGGGTGGGGCAGGCACTCTGTAGCTGAGGAAGTGG
GGCCCGACAGC
>GOT11:
GATGCTGAAATAACTGCTACCTGACTTCAAATCAAGTGCTTTAGTGACTCCAGCCAGCTGTGAGATCACG
GCAGTTCTGGACAGTCATAAGATTATGGGAGGAGCAGAGGGGGAAAGTGCAATCTACGAGGTGCTGA
CCCAGGGCCATCAGAACTGTGAGGGTGATTGGGTTTCCTGGGTTCTGATGTATCAGGGACCCTCATGGG
AGTGGGCTGGGCTTGGGTGCCAATGTGTCTGTAGGACAGCCACAGACAGGAAGTCTGACATCTAGAAC
TGGGGAATACACACCATGCAAGAGCTGCCTAAGACACATACCAGGGAGGATATTACACACTCTTGGTGT
GGCCATTCACCCTGACCCTAGAAACCCTGCTTGGGCTTGGCACCCCTGCAAGAAAACCCCACCACTACTT
CCTGGCAACTTTTATTTCATCTCAAAAGAGAGAGAACCCCACTTGTCC
>OT1 (another primer set to verify GUIDE-Seq data)
CAGAAGAGGTTGTTGGATTCCCTGGGACTGGAGTTGAGGTGGTGGTGAGTCACGGTGAACTGGGGTCC
TCTGTAGAAGAGCAGCGGGTGTCCTTAATTGCTGAGCTCTCTCCAGCCCTAACGGCCGCATCAGTTTGGC
TAGGCTGGCACTCAGTGAGGTCCAGGGATCTGCCTGTCTCTTGCCCCAGCGCTGGAGTTACAGACTCAC
ACTGTCATCCCTCGCTTTTGTGTGGATTCTGGGATCCAAACTCAGTCTTCTTGCTTTTATGGTAGTTGCCTT
GCCGAGTGAGTGCCTCTCAGTTCCTGGCATTTCTGCTCCAGTTGTACAATCCTTCCTGCTGTTTCTGATCT
GGAGGCGTTAGAGCTGCCTCCCCTGCTTTATTTGTCCTGCCCGGGCTAGTTTGGTGTTTCCTGACTGTTCT
TAATTGGCTTTCAAGTCATGTGACCTAGACTCCTGATAGAAATAACCCAAGGAAGGAGCGTTTATTTGGA
CTGACAGTTTGGAG

Nature Biotechnology: doi:10.1038/nbt.3471
